# Supplementary material for: Non-Invasive Monitoring of Cutaneous Wound Healing in Non-Diabetic and Diabetic Model of Adult Zebrafish Using OCT Angiography
Source: Bioengineering (Basel). 2023 Apr 27;10(5):538. doi: 10.3390/bioengineering10050538 (PMC10215851; doi:10.3390/bioengineering10050538)
Supplement: Supplementary file 1 [file bioengineering-10-00538-s001.zip › bioengineering-2346801-supplementary.pdf]

bioengineering

Article

Non-Invasive Monitoring of Cutaneous Wound Healing in Non-Diabetic and Diabetic Model of Adult Zebrafish Using OCT Angiography

Jaeyoung Kim [1,2,3,†], Suhyun Kim [4,5,†] and Woo June Choi [6,*]

[1] Research Institute for Skin Image, Korea University College of Medicine, Seoul 08308, Republic of Korea; jaykim830@gmail.com
[2] Department of Dermatology and Skin Science, University of British Columbia, Vancouver, BC V6T 1Z1, Canada
[3] Departments of Cancer Control Research and Integrative Oncology, British Columbia Cancer Agency, Vancouver, BC V5Z 1L3, Canada
[4] Department of Biomedical Sciences, Korea University College of Medicine, Seoul 02841, Republic of Korea; dieslunae@naver.com
[5] Zebrafish Translational Medical Research Center, Korea University Ansan Hospital, Ansan 15355, Republic of Korea
[6] School of Electrical and Electronics Engineering, Chung-Ang University, Seoul 06974, Republic of Korea
* Correspondence: cecc78@cau.ac.kr
† These authors contributed equally to this work.

Supplementary Materials

(a)

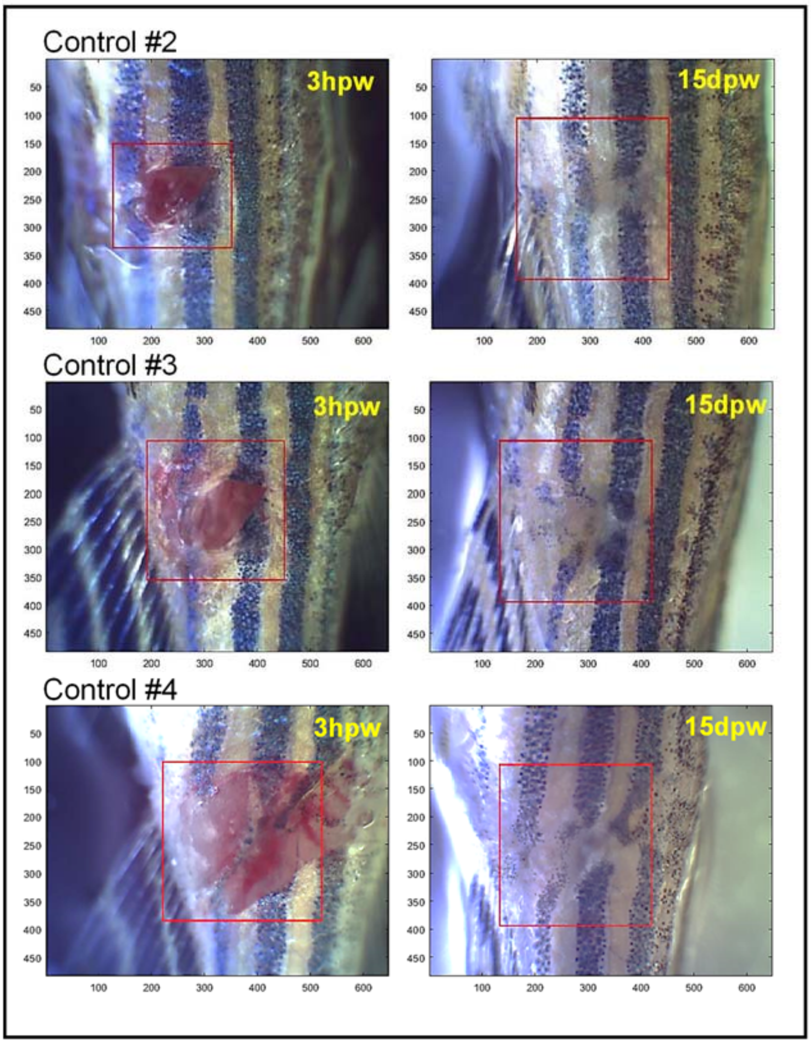
Control #2
3hpw
15dpw
Control #3
3hpw
15dpw
Control #4
3hpw
15dpw

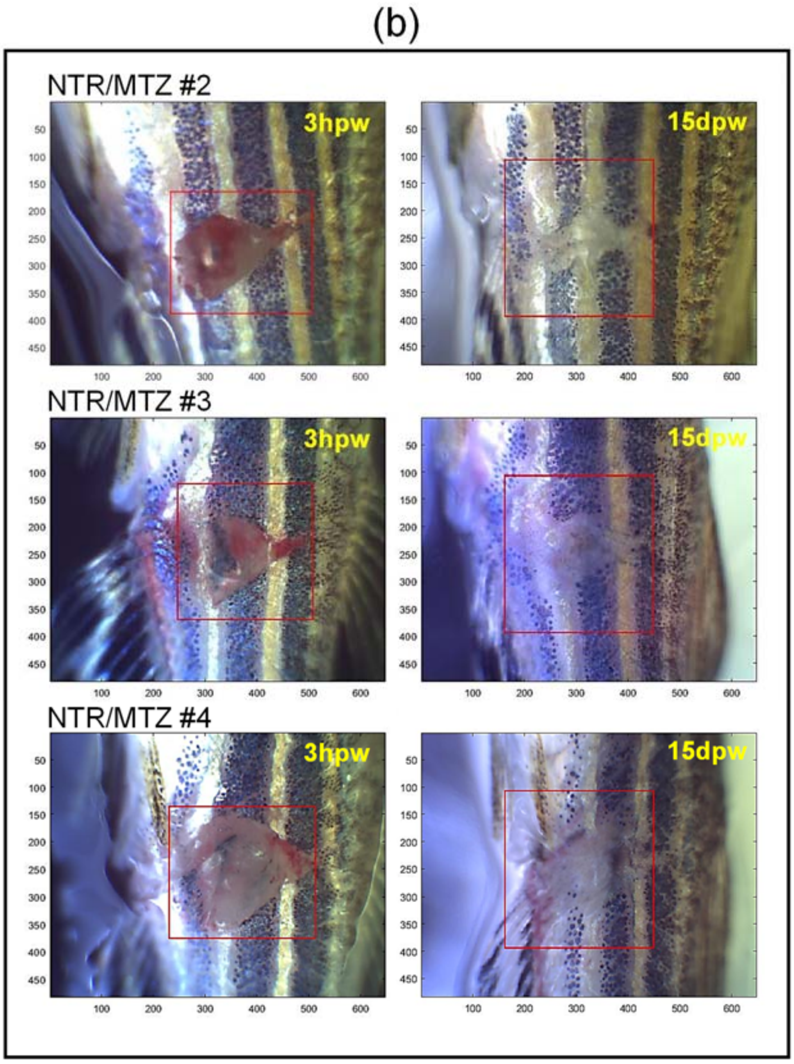

Figure S1. Wound healing of three adult zebrafishes in control (normal) group (a) and diabetic model group (b). The superficial views exhibit the repair of wounds in red boxes at 3 hours and 15 days post wounding, respectively. Visually, the regeneration of the stripe patterns is less for the diabetic fishes than those on control ones.
